# Evaluation of the Effects of Covering With Polyglycolic Acid Sheet on Wound Healing: A Pilot Histopathological Study

**DOI:** 10.7759/cureus.27209

**Published:** 2022-07-24

**Authors:** Yasumasa Kakei, Kazunobu Hashikawa, Kaito Uryu, Ryuichiro Funahara, Manabu Shigeoka, Masaya Akashi

**Affiliations:** 1 Department of Oral and Maxillofacial Surgery, Kobe University Hospital, Kobe, JPN; 2 Department of Plastic Surgery, Nagoya University Graduate School of Medicine, Nagoya, JPN; 3 Department of Oral and Maxillofacial Surgery, Kobe University Graduate School of Medicine, Kobe, JPN; 4 Division of Pathology, Department of Oral and Maxillofacial Surgery, Kobe University Graduate School of Medicine, Kobe, JPN

**Keywords:** ionized calcium-binding adapter molecule 1, masson’s trichrome stain, α-smooth muscle actin, wound healing, polyglycolic acid sheet

## Abstract

Purpose: Although the usefulness of polyglycolic acid (PGA) sheet for wound dressing has been recently reported, its histopathological effect on wound healing is not completely elucidated. This pilot study focused on the neo-epithelium formation and the remaining inflammation.

Methods: Full-thickness defects of 8 mm were created on the back of seven-week-old rats. Four rats were divided into the control (raw surface) group and the PGA group, in which the wounds were covered with a PGA sheet. The wounds were assessed on days seven and 12 after wound creation. The length of neo-epithelium on day seven was measured by referring to Masson’s trichrome (MT) and α-smooth muscle actin (α-SMA) staining. The remaining inflammation on days seven and 12 was assessed with ionized calcium-binding adapter molecule 1 (Iba-1) staining.

Results: The average values of neo-epithelium length on day seven measured by referring to the borderline between MT staining and α-SMA expression were 959.2 μm in the control group and 582.2 μm in the PGA group. The number of Iba-1-positive cells on day 12 was significantly higher in the PGA group than in the control group.

Conclusions: To assess the neo-epithelium length and the remaining inflammation, the α-SMA, MT, and Iba-1 staining may be appropriate.

## Introduction

Polyglycolic acid (PGA) sheets are suture-reinforcement materials absorbed within 4-15 weeks [1]. Recently, the clinical use of PGA sheets for preventing delayed perforation and closure of a fistula in open and endoscopic surgery [1,2] or covering wounds following partial glossectomy [3,4] has been reported. However, few histopathological studies have focused on the effect of PGA sheet covering on wound healing.

Kibe et al. [5] focused on the effect of PGA sheet covering on early wound healing, specifically on days four and seven after wound creation on the back of rats, and reported that PGA sheet covering suppressed contracture and reduced expression of α-smooth muscle actin (α-SMA, a marker of myofibroblasts, the shrinking of which causes scar contracture). They also reported that PGA covering did not affect the length of the neo-epithelium at the wound site [5]. In another animal experiment by Inokuchi et al. [6], wounds with or without PGA sheets and fibrin glue at one, two, four, and eight weeks after partial tongue resection in rabbits were histologically evaluated. They found that the wounds of the control and PGA groups exhibited partial repair of the neo-epithelium after two weeks. Four weeks after tongue resection in rabbits, epithelization was almost complete in the control and PGA groups, and PGA was completely absorbed or detached. The histological features of the specimens in postoperative week eight were similar to those in week four. Interestingly, they found residual PGA fibers and macrophages in the PGA wound two weeks postoperatively, whereas collagen fibers were found toward the center of the control wound. No infiltration of inflammatory cells was found at postoperative week four in either group.

As mentioned above, the formation of neo-epithelium and evaluation of residual inflammatory cells may be adequate as an index to evaluate the effect of PGA sheet covering on wound healing. However, in previous reports, the effect of PGA sheet covering on wound healing was histologically evaluated using only hematoxylin and eosin (HE) staining. The current study showed that the combination of α-SMA and Masson’s trichrome (MT) staining was adequate for evaluating the neo-epithelium and ionized calcium-binding adapter molecule 1 (Iba-1, a marker of microglia and macrophages) staining was adequate for evaluating residual inflammation within 14 days after wound creation.

## Materials and methods

Seven-week-old female Wistar rats (CLEA Japan, Inc., Tokyo, Japan) were used in the current study. All animal experiments were performed in accordance with the Guidelines for Animal Experimentation at Kobe University Animal Experimentation Regulations (permission number: P180501) and were approved by the Institutional Animal Care and Use Committee.

Surgical wounds were created on the back of the rats, according to a previous report [5]. Briefly, rats were anesthetized with an intraperitoneal injection of medetomidine (0.15 mg/kg), midazolam (2 mg/kg), and butorphanol (2.5 mg/kg). The hairs on the back of the rats were shaved with a razor, and the skin was disinfected with 80% ethanol. Two circular 8-mm full-thickness wounds were made on the dorsal skin of the rats using a disposable biopsy punch (Kai Medical, Tokyo, Japan). The wounds were covered with a PGA sheet (Neoveil Nano®️, Gunze Co., Ltd., Tokyo, Japan) and fibrin glue (Bolheal®️, Chemo-Sero-Therapeutic Research Institute, Kumamoto, Japan) and fixed using a rat jacket [5]. In the control rats, the wounds were not covered with any dressing. The rats were housed in individual cages. Four rats were used in this experiment. Two rats with a raw surface and PGA covering were sacrificed seven and 12 days after wounding. Eight specimens were assessed histopathologically. The harvested dorsal skin specimens were fixed with formalin and embedded in paraffin. The sections were stained with HE (A1), MT, mouse monoclonal anti-human α-SMA (product code: M0851, 1:150, Agilent, Santa Clara, CA, USA), and rabbit monoclonal antibody against Iba-1 (product code: ab178846, 1:2,000, Abcam, Cambridge, UK). The length of the neo-epithelium was measured using MT and α-SMA staining, as mentioned below. Eight wound edges (i.e., four edges in the control group and PGA group each, on day seven) were used for neo-epithelium measurements. In accordance with previous studies [7,8], the number of macrophages was counted in subepithelial areas up to 1000 µm from the raw surface. Five independent high-power fields (×400) were selected for eight specimens, and the mean number was calculated. Iba-1-positive round cells were identified as macrophages. Statistical significance was analyzed using SPSS Statistics version 22 (IBM Corp., Armonk, NY, USA). A comparison of the number of Iba-1-positive cells was done using paired or Student’s t-tests. In this study, a value of p < 0.05 indicated statistical significance.

## Results

**Figure 1:**
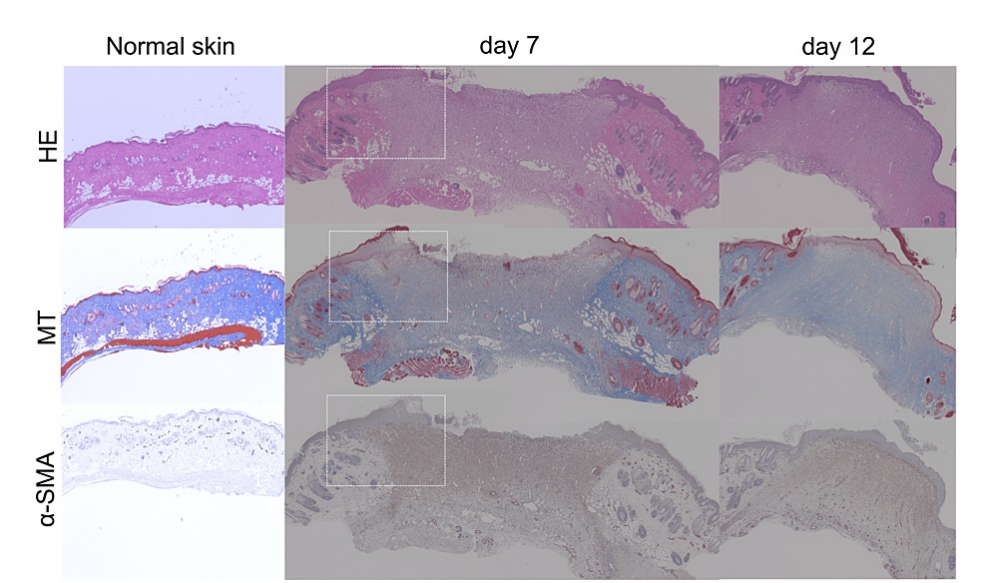
Hematoxylin and eosin (HE), Masson’s trichrome (MT), and α-smooth muscle actin (α-SMA) staining Left panels: HE, MT, and α-SMA staining of the normal dorsal skin of rats. Center panels: Raw surface seven days after wound creation. Dotted white boxes indicate the wound edges. Right panels: Raw surface 12 days after wound creation.

**Figure 2:**
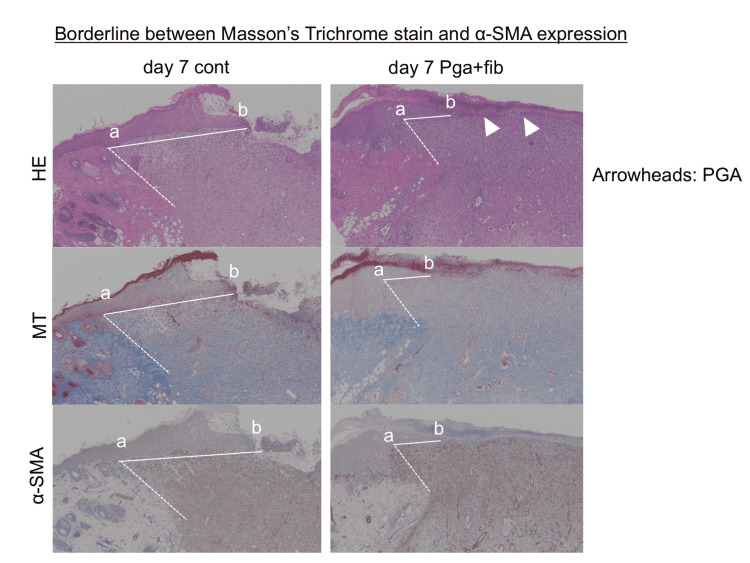
The borderline between MT stain and α-SMA expression The borderline between MT staining and α-SMA expression. Left panels: Control wounds seven days after wound creation. Right panels: Wound covered with a polyglycolic acid (PGA) sheet and fibrin glue (fib) seven days after wound creation. (a-b) indicates the length of the neo-epithelium. HE: hematoxylin and eosin; MT: Masson’s trichrome; α-SMA: α-smooth muscle actin.

Figure 1 shows HE, MT, and α-SMA staining of the dorsal skin of the rats. Seven days after wound creation, the deposition of collagen fibers was observed with MT staining, and the accumulation of myofibroblasts was observed with α-SMA staining. The neo-epithelium could be defined as the epithelium lining the areas with the deposition of collagen fibers and the accumulation of myofibroblasts. Therefore, the length of the neo-epithelium could be more precisely evaluated by measuring the distance between the edge of the epithelium and the borderline of MT staining and α-SMA expression, as shown in Figure 2. By referring to the borderline of MT staining and α-SMA expression, the average values of neo-epithelium were 959.2 (±164.3) μm in the control group and 582.2 (±443.0) μm in the PGA group.

**Figure 3:**
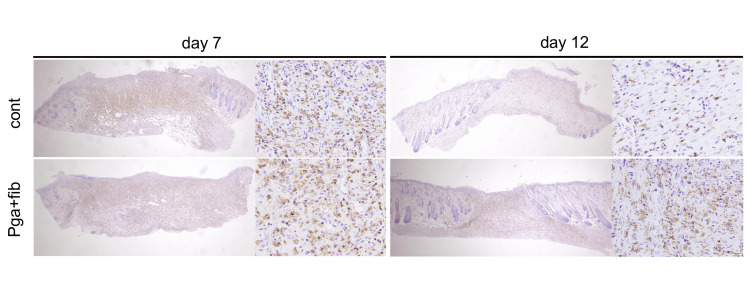
Staining of ionized calcium-binding adapter molecule 1 (Iba-1) Scale bar: 500 μm. cont: control; PGA: polyglycolic acid; fib: fibrin glue.

**Figure 4:**
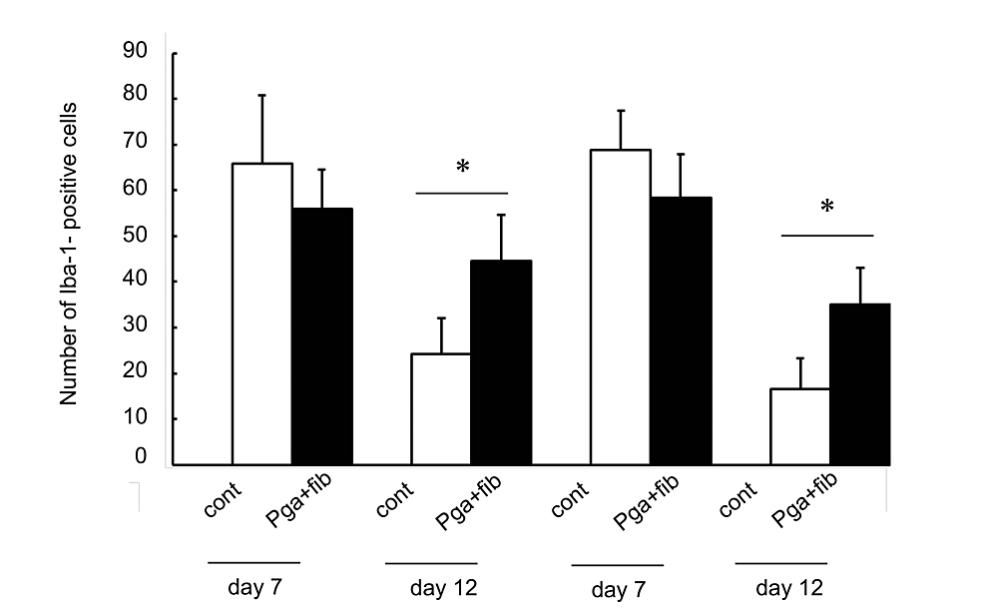
The number of Iba-1-positive cells * A value of p < 0.05 was considered statistically significant. Iba-1: ionized calcium-binding adapter molecule 1; cont: control; PGA: polyglycolic acid; fib: fibrin glue.

While evaluating the effect of PGA covering on wound healing, histopathological assessment of not only the neo-epithelium but also inflammation is important. Iba-1 staining shows the accumulation of Iba-1-positive cells in the wounded areas seven days after wound creation (Figure 3). The number of Iba-1-positive cells after seven days in wounds covered with PGA was lower than that seen in the control wounds at seven days; however, the difference was not significant. In contrast, the number of Iba-1-positive cells at 12 days in wounds covered with PGA was significantly higher than that seen in the control wounds at 12 days (Figure 4).

## Discussion

The current pilot study aimed to evaluate the effects of PGA sheet covering on wound healing. Neo-epithelium formation is a critical process in wound healing. Therefore, it should be precisely evaluated. PGA sheets are absorbable; however, they are foreign to the human body. Recently, two minor complications (granuloma-like neoplasm and abnormal postoperative bleeding) have been reported following the PGA sheet covering for wounds after oral cancer resection [7]. To promote safer clinical applications of PGA, histopathological examination of the biological response to PGA is essential.

A previous study by Kibe et al. [5] reported that the measured values of the neo-epithelium length were approximately 500 μm in the control and PGA groups on day four and approximately 1000 μm on day seven. PGA covering did not significantly affect the length of the neo-epithelium at the wound site on day seven [5]. However, they noted that epithelial cells at the wound margins extend along the bottom of the wound when a foreign body or other unnecessary organization exists on the wound surface [5]. In this study, although the number of experiments was small, the neo-epithelium length was shorter in the PGA group than in the control group.

The combination of MT and α-SMA staining can indicate the border between the deposition areas of coarse collagen fibers and the areas of myofibroblast accumulation. A previous study reported that MT staining of wounded skin provides an understanding of the wound healing process, such as the migration and reorganization of collagen fibers in the healed skin at every wound healing stage [9]. MT staining also enables the differentiation of fine and coarse collagen fibers, which usually appear in the remodeling phase of wound healing and influence scar formation [9]. In circulatory diseases, the combination of MT and α-SMA staining can differentiate between subendocardial fibrosis (MT) and the subendocardial smooth muscle layer (α-SMA) [10].

Interestingly, although the difference was not significant, the number of Iba-1-positive cells was lower in the PGA group than in the control group. The process of wound healing can be divided into the initial inflammatory, proliferative, and remodeling phases [11-14]. The initial inflammatory phase starts from injury occurrence and ends within seven days of injury occurrence [12]. Although significant differences were not found, a PGA sheet on the wound surface may reduce inflammation in the initial inflammatory phase compared with the raw surface; however, further studies are required.

Although the number of experiments was small, the number of Iba-1-positive cells was significantly higher in the PGA group than in the control group 12 days after wound creation. Granuloma-like neoplasm has been reported as a minor complication following tongue resection and PGA covering. Okuyama et al. [15] indicated that granuloma-like neoplasms could be an inflammatory source, and the main factor in the onset may be induced by PGA sheets. The number of macrophages increased, reached a maximum concentration seven days after injury, and declined 14 days after injury [12]. Further studies focusing on the effect of PGA covering on residual inflammation at 14 days or more after wound creation are required.

## Conclusions

To evaluate the effect of PGA covering on wound healing, a combination of MT and α-SMA staining is appropriate for measuring the neo-epithelium length. A PGA sheet on the wound surface may reduce inflammation in the initial inflammatory phase compared with the raw surface, but may cause persistence of inflammation. Iba-1 staining is appropriate for assessing residual inflammation.
